# No Evidence for Early Modulation of Evoked Responses in Primary Visual Cortex to Irrelevant Probe Stimuli Presented during the Attentional Blink

**DOI:** 10.1371/journal.pone.0024255

**Published:** 2011-08-25

**Authors:** Oscar Jacoby, Troy A. W. Visser, Bianca C. Hart, Ross Cunnington, Jason B. Mattingley

**Affiliations:** 1 Queensland Brain Institute, University of Queensland, Brisbane, Queensland, Australia; 2 School of Psychology, University of Queensland, Brisbane, Queensland, Australia; University of Minnesota, United States of America

## Abstract

**Background:**

During rapid serial visual presentation (RSVP), observers often miss the second of two targets if it appears within 500 ms of the first. This phenomenon, called the *attentional blink* (AB), is widely held to reflect a bottleneck in the processing of rapidly sequential stimuli that arises after initial sensory registration is complete (i.e., at a relatively late, post-perceptual stage of processing). Contrary to this view, recent fMRI studies have found that activity in the primary visual area (V1), which represents the earliest cortical stage of visual processing, is attenuated during the AB. Here we asked whether such changes in V1 activity during the AB arise in the initial feedforward sweep of stimulus input, or instead reflect the influence of feedback signals from higher cortical areas.

**Methodology/Principal Findings:**

EEG signals were recorded while participants monitored a sequential stream of distractor letters for two target digits (T1 and T2). Neural responses associated with an irrelevant probe stimulus presented simultaneously with T2 were measured using an ERP marker – the C1 component – that reflects initial perceptual processing of visual information in V1. As expected, T2 accuracy was compromised when the inter-target interval was brief, reflecting an AB deficit. Critically, however, the magnitude of the early C1 component evoked by the probe was not reduced during the AB.

**Conclusions/Significance:**

Our finding that early sensory processing of irrelevant probe stimuli is not suppressed during the AB is consistent with theoretical models that assume that the bottleneck underlying the AB arises at a post-perceptual stage of processing. This suggests that reduced neural activity in V1 during the AB is driven by re-entrant signals from extrastriate areas that regulate early cortical activity via feedback connections with V1.

## Introduction

Our capacity to process information about the external environment is restricted to only a small portion of the inputs provided by our sensory organs [1]. To make best use of limited information processing resources, mechanisms of selective attention prioritise and enhance the processing of some stimuli at the expense of others [2]. For example, when participants are cued to attend to a specific visual location, behavioural responses to target stimuli presented at that location tend to be faster than responses to stimuli presented at other spatial locations [3]. A similar compromise is evident in the time domain when participants must identify two target stimuli presented within about 500 ms of each other. Here, participants' ability to identify the second target (T2) is impaired, relative to when the two targets are separated by a longer interval, or when the first target (T1) does not have to be identified (the attentional blink, AB; [4], [5]). Thus, processing of T1 seems to be selectively prioritised at the expense of T2 processing.

A question that has been the focus of much cognitive neuroscientific research is whether such temporal limits in attention arise during initial perceptual processing in early visual areas, or at later post-perceptual stages such as response selection or the updating of working memory [1]. Most existing findings support the post-perceptual view, but a recent fMRI study that found attenuated activity in the primary visual cortex (area V1) during the AB [6] raises the possibility of an early perceptual effect as well. Here we exploited the high temporal resolution of EEG to determine whether the earliest visual evoked response in V1, which occurs less than 100 ms after target onset, is indeed altered during the AB, or whether V1 activity is influenced later in processing by feedback from extrastriate areas.

Neurophysiological studies of spatial selection suggest that preferential processing of stimuli at cued or attended spatial locations can arise very early in the cortical processing pathway. Functional magnetic resonance imaging (fMRI) studies have demonstrated that spatially attended stimuli evoke larger blood oxygen level dependent (BOLD) responses than unattended stimuli in area V1, both when attention is directed voluntarily to a region of space [7], [8], or when it is captured by an onset cue [9], [10]. Complementary evidence has been provided by a recent event-related potential (ERP) study by Kelly, Gomez-Ramirez and Foxe [11]. This study found that the C1, a component of the ERP believed to reflect the initial feedforward sweep of activity through V1 [12], was larger for stimuli presented at attended relative to unattended locations. Later ERP components believed to reflect perceptual processing in extrastriate cortex (P1 and N1; [12]), are also larger for stimuli at attended relative to unattended locations [13]–[16]. Thus, the combined evidence from fMRI and ERP methodologies indicates that spatial selection can bias processing from the very first stages of visual analysis in V1.

In contrast to spatial selection, limits in temporal attention are widely held to arise at relatively late, post-perceptual stages of processing. Vogel, Luck and Shapiro [17, Experiment 1], for example, found that the amplitude of the P1 and N1 components of the ERP did not vary with the asynchrony between T1 and T2 items in an AB task. They had participants search for two target items embedded within a rapid serial visual presentation (RSVP) stream of distractors. The serial position of T2 relative to T1 within the stream (referred to as ‘lag’) was varied between trials. ERPs evoked by an irrelevant probe stimulus presented simultaneously with T2 were measured separately for each lag. Participants were poorer at identifying T2 when it was presented during the AB period than when it was presented outside the AB. Despite this behavioural impairment, the amplitude of the P1 and N1 components evoked by the probe accompanying T2 did not vary as a function of lag, suggesting perceptual processing remained intact throughout the AB (see also [18]). In a separate experiment, Vogel et al. [17, Experiment 4] measured the extent to which T2 stimuli evoked a P3, an ERP component implicated in a variety of post-perceptual processes including the updating of working memory [19], [20], conscious awareness [21], and response selection/execution [22], [23]. The amplitude of the P3 component evoked by T2 was significantly smaller when T2 was presented during the AB than outside it, suggesting post-perceptual processes underlie the behavioural deficits observed during the AB (see also [18], [24]–[28]).

Results from fMRI studies that have examined the neural correlates of the AB are somewhat less clear-cut. Consistent with a post-perceptual locus for the deficit, Marois, Yi and Chun [29] found that T2 scene stimuli presented during the AB evoked BOLD responses in a region of visual cortex that responds selectively to scenes (the parahippocampal place area, PPA; [30]) even when they were not correctly identified. However, BOLD responses in parietofrontal regions implicated in the allocation of attentional resources to visual stimuli [31],[32] were only evoked by correctly identified T2 stimuli (see also [33]). These findings strongly implicate post-perceptual processes as the source of capacity limitations underlying the AB.

Apparently at odds with this suggestion, however, are the findings of a recent fMRI study by Williams, Visser, Cunnington and Mattingley [6] which measured BOLD responses in V1 evoked by T2 stimuli presented during the AB. Such a task is complicated by poor temporal resolution of fMRI, as BOLD responses evoked by T2 stimuli are difficult to disentangle from those evoked by neighbouring items within the RSVP stream. However, to overcome this difficulty, Williams et al. [6] presented T2 at a different location than other items in the RSVP stream. Due to the retinotopic organisation of the primary visual cortex, stimuli presented at different visual field locations will activate different regions within V1. Thus, Williams et al. were able to isolate T2-related activity in V1 by restricting their analyses to regions responsive to the T2 locations used in their study. In contrast to the suggestion that perceptual processing remains intact during the AB [17], [18], [29], [33], Williams et al. found that BOLD responses to T2 stimuli in V1 were substantially reduced when T2 was presented during- relative to outside- the AB. Similar results were reported in a study by Hein, Alink, Kleinschmidt and Muller [34], who found that BOLD responses in early visual cortex were reduced when T2 stimuli presented during the AB were incorrectly identified, relative to when they were correctly identified.

A possible reconciliation of these contrasting findings is provided by re-entrant feedback models of perception [35]–[39], which argue that perception consists not only of a feedforward sweep of information from lower perceptual areas to higher regions of cortex, but also of feedback signals from higher back to lower areas of cortex. These models are supported by anatomical studies demonstrating axonal tracts extending in both directions between higher and lower areas in the primate visual system [40], and also by electrophysiological studies suggesting that spatial attention-related activity in extrastriate areas might occur earlier than in V1 [41], [42]. Consideration of the re-entrant framework opens up the possibility that AB-related modulations of V1 measured with fMRI [6], [34] reflect re-entrant signals from capacity-limited extrastriate areas back to V1, consistent with a post-perceptual locus of the AB, rather than a suppression of the initial feedforward sweep of information through V1. These two options cannot be disentangled directly on the basis of fMRI data due to their rather low temporal resolution. Although the ERP study by Vogel et al. [17] indicated that early extrastriate activity evoked by T2 (as reflected by the P1 and N1 components) is not suppressed during the AB, it remains possible that even earlier (<100 ms) responses in V1 are affected independently.

To address this possibility, we used ERPs to compare the amplitude of the C1 component – an ERP marker that reflects feedforward V1 activity – evoked by stimuli presented during and outside the AB. Previous ERP studies of the AB [17], [18], [27] were unable to measure this component, as the stimuli used in these studies were presented at fixation, a location at which the C1 tends to be weak or entirely absent from the ERP due to the anatomical organisation of V1 [43], [44]. We overcame this difficulty by measuring the C1 evoked by a task-irrelevant probe presented simultaneously with T2, at a peripheral visual field location found to evoke a reliable C1 component [12]. If initial V1 activity is altered for stimuli presented during the AB, we would expect to observe a reduced amplitude in the C1 component. By contrast, if V1 responses are only modulated by re-entrant feedback signals late in visual processing, the C1 component should remain consistent across lag. To anticipate, we obtained a robust AB in behavioural testing, and a reliable C1 component for the irrelevant probe presented with T2, but found that the amplitude of the C1 component was unaltered during the AB.

## Methods

### Participants

Twenty-seven adult volunteers (13 male, 14 female, aged 19 – 27 years) took part in the present study in exchange for an honorarium of $10 AUD per hour. All participants had normal or corrected-to-normal vision.

### Ethics Statement

All experimental procedures were conducted in accordance with the principles expressed in the Declaration of Helsinki, and were approved by the University of Queensland Ethics Committee. Written informed consent was obtained from each participant prior to participation.

### Apparatus and Stimuli

Stimuli were presented against a mid-grey background (RGB coordinates 128, 128, 128) on a 21 inch CRT monitor (NEC, Accusync 120) at a screen resolution of 1152×864 pixels and a 100 Hz refresh rate. A viewing distance of 72 cm was maintained using a chin rest. Stimulus presentation and response recording was controlled using Presentation software (Presentation 13.0, Neurobehavioral Systems), running on a Pentium IV 3GHz desktop computer. The centrally presented RSVP stream for each trial consisted of two target digits interspersed among upper-case letter distractors, presented in ‘Arial’ font and subtending 0.7° of visual angle vertically and between 0.3° and 0.6° of visual angle horizontally. Distractor letters were randomly selected without replacement from the English alphabet (excluding the letters I, O, Q and Z). The first target (T1) was either a 2 or a 5, and the second target (T2) was either a 3 or an 8, with both targets randomly selected on each trial. All items within the RSVP stream were presented in white (RGB coordinates 255, 255, 255), except T2, which was presented in light grey (RGB coordinates 170, 170, 170) to increase task difficulty.

**Figure 1:**
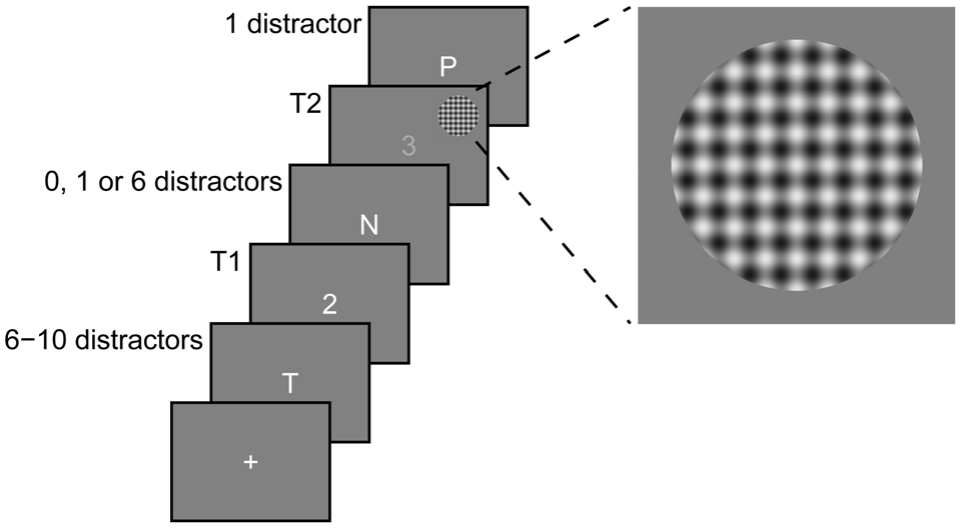
Schematic representation of the sequence of displays in the RSVP stream. The two target digits (T1 and T2) were embedded within a stream of distractor letters. Lag was manipulated by varying the number of distractor letters between the target digits. On half of the trials presented at each lag, an irrelevant probe stimulus was presented simultaneously with T2 in the upper-right quadrant of the display.

A task-irrelevant probe stimulus (see Figure 1) was presented simultaneously with T2 on half of the trials for each lag. This circular stimulus consisted of a black and white checkerboard pattern with a diameter of 2° of visual angle. The checkerboard pattern was created using Matlab (Version 7.6, MathWorks) by overlapping vertical and horizontal sinusoidal gratings with a spatial frequency of 4 cycles per degree of visual angle and a peak contrast of 80%. When present, the centre of the probe was located 4° from fixation at a polar angle of 25° above the horizontal meridian. Previous research [12] and pilot investigations in our own laboratory have demonstrated that presenting this stimulus at this location evokes a reliable C1 component in most participants.

### Procedure

The general structure of the RSVP stream for each trial is illustrated in Figure 1. Each trial began with a central fixation cross, which remained until the participant pressed the space bar on a computer keyboard. This initiated the RSVP stream. Consistent with previous ERP studies of the AB (e.g., [17]), each RSVP item was presented for 20 ms with an 80 ms blank display between adjacent items. The number of letters preceding T1 varied randomly between 6 and 10 on each trial so participants could not predict the onset of T1. The first and second targets were separated by an additional 0, 1, or 6 distractors (i.e., lags 1, 2, and 7). Lag was chosen randomly on each trial with the proviso that each lag occurred equally often during a block of trials. A final distractor letter was presented after T2 which acted as a backward mask [35]. A 500 ms blank screen delay followed the RSVP stream, after which participants were prompted to report the identity of T1 (the text “2 ? 5” appeared on screen) by pressing buttons on a computer keyboard. Once they had made their response to T1, they were prompted to identify T2 (the text “3 ? 8” appeared on screen). Both responses were unspeeded. Once both responses had been entered, the central fixation cross re-appeared, and participants pressed the spacebar to initiate the next trial.

Participants were fitted with a 64-electrode EEG cap, and asked to avoid head, eye and body movements during the tasks. Six practice trials were completed during which on-screen feedback was provided. Participants completed 18 blocks of 48 trials, each containing an equal number of trials for each of the six conditions created by the crossed factors of lag (1, 2, and 7) and probe condition (present, absent). In total, 144 trials were presented for each condition. The lights in the laboratory were turned off during the testing blocks to minimise potential distractions from extraneous visual stimuli within the room. Participants were encouraged to take rest breaks between blocks to avoid fatigue. The entire procedure took approximately 2 hours per participant.

### EEG Recording

Continuous EEG data were recorded using a BioSemi Active Two system (BioSemi, Amsterdam, Netherlands), digitized at a 1024 Hz sample rate with 24-bit A/D conversion. The 64 active scalp Ag/AgCl electrodes were arranged according to the international standard 10–10 system for electrode placement [45] using a nylon head cap. During recording, all scalp electrodes were referenced to the standard BioSemi reference electrodes. Eye movements were monitored using bipolar horizontal electrooculographic (EOG) electrodes placed at the outer canthi of each eye, and bipolar vertical EOG electrodes placed above and below the left eye.

### EEG Data Analysis

Offline EEG data analysis was performed using Brain Electrical Source Acquisition (BESA 5.3; MEGIS Software GmbH, Gräfelfing, Germany). The data for the scalp electrodes were re-referenced to the average of all 64 scalp electrodes and subjected to Low-pass (0.1 Hz, 6dB/oct, forward shift) and high-pass (45 Hz, 12db/oct, zero phase shift) digital filters. Noisy channels, identified by visual inspection of the data, were interpolated. The data were then segmented into epochs from 100 ms before to 300 ms after T2 onset, with the average voltage in the 100 ms prestimulus interval serving as a baseline. Epochs in which the difference between the maximum and minimum voltage exceeded 120 µV at any channel were automatically rejected to remove epochs contaminated by blinks, eye movements and other artifacts. An average of 4% of trials were rejected for violating this criterion. Trials with incorrect T1 responses (7%) were also excluded from the ERP analyses, on the grounds that the source of error on these trials is unknown. Averaged waveforms were then created for each level of lag (1, 2, and 7), separately for each probe condition (present, absent). To isolate activity evoked by the probe stimulus, a *difference waveform* [17], [46] was created for each level of lag by subtracting the waveform evoked when the probe was absent from the waveform evoked when the probe was present. Analyses of the ERPs evoked by the probe stimulus were conducted on these difference waveforms.

## Results

All statistical tests were conducted using SPSS, with a two-tailed alpha level of.05. Mauchly's Test of Sphericity was applied to all within-subjects F tests. Greenhouse-Geisser epsilon adjustments were made to degrees of freedom for these F tests wherever the assumption of sphericity was untenable. Unadjusted degrees of freedom are reported for all F tests. Data from two individuals who performed at chance level on T2 across all lags were excluded from all analyses.

### Behavioural Results

Mean T1 identification accuracy was submitted to a 3×2 within-subjects ANOVA with factors of lag (1, 2, and 7) and probe condition (present, absent). This analysis revealed no significant main effect of lag, *F*(2, 48) = 2.22, ε = .78, *p* = .119, η_p_^2^ = .085, no effect of probe condition, *F*(1, 24) = 0.95, *p* = .340, η_p_^2^ = .038, and no lag × probe condition interaction, *F*(2, 48) = 0.33, *p* = .722, η_p_^2^ = .013, indicating that T1 identification did not vary as a function of lag or probe condition.

**Figure 2:**
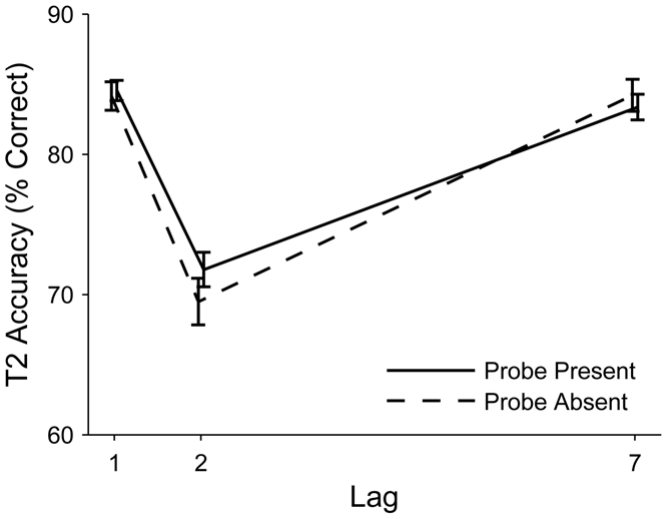
Mean percentage of correct T2 responses as a function of lag, plotted separately for each probe condition. Error bars represent the within-subjects standard error of the mean.

Mean T2 identification accuracy (calculated only on trials in T1 was identified correctly) was also submitted to a 3×2 within-subjects ANOVA with factors of lag and probe condition (see Figure 2). A significant main effect of lag on T2 accuracy, *F*(2, 48) = 34.88, ε = .69, *p*<.001, η_p_^2^ = .592, was followed up with paired-sample *t*-tests. In line with the typical behavioural pattern of performance for T2 in the AB [47], accuracy was significantly lower at lag 2 (*M* = 70.63%, *SD* = 11.56%) than it was at lag 1 (*M* = 84.34%, *SD* = 13.82%), *t*(24) = 6.84, *p*<.001, or lag 7 (*M* = 83.78%, *SD* = 15.36%), *t*(24) = 5.82, *p*<.001. T2 accuracy did not differ between lag 1 and lag 7, *t*(24) = 0.51, *p* = .613. There was no main effect of probe condition and no interaction between the factors, indicating that the effect of lag on T2 accuracy was not modulated by the presence of the probe.

### ERP Results

Three primary ERP components (C1, P1, and N1) evoked by the probe stimulus used in this study were identified on the basis of visual inspection of grand average waveform topography maps, in conjunction with comparisons with the ERP results of previous research by Di Russo et al. [12], which used an identical visual stimulus. For each component, the mean amplitude across a 30 ms time window encapsulating the waveform peak in the grand average was calculated across a cluster of electrodes at which the component was maximal. These mean amplitudes were then subjected to two-way within-subjects ANOVAs with factors of lag and electrode.

**Figure 3:**
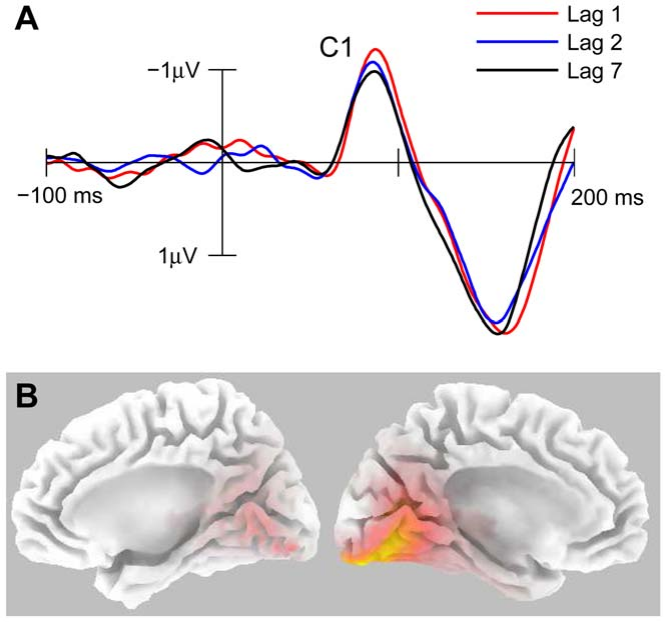
ERP analysis of the C1 component evoked by T2. (A) Grand average difference waveforms collapsed across electrodes POz, PO4, PO8, Oz, and O2, shown separately for each lag condition. Note that negative is plotted upwards. (B) Medial views of the two cortical hemispheres depicting the estimated current density distribution calculated by sLORETA for the activity observed in the grand average difference waveforms (collapsed across lags) at the peak of the C1 component (87 ms). The regions sLORETA identified as most active are presented in yellow, and fall along the banks of the calcarine fissure in the left hemisphere, corresponding to area V1.

The C1 component was measured as the mean amplitude between 70 and 100 ms post stimulus onset at a cluster of five posterior occipital electrodes (POz, PO4, PO8, Oz, and O2). As in the study by Di Russo et al. [12], the C1 component for the probe stimulus was a negative voltage deflection maximal at the PO4 electrode at ∼87 ms post stimulus. The peak corresponding to the C1 component is identified in Figure 3A, which depicts a plot of the difference waveforms associated with the probe stimulus separately for each lag, collapsed across the five analysed electrodes. The 3 (lag)×5 (electrode) within-subjects ANOVA on mean C1 amplitudes revealed no significant main effect of lag, *F*(2, 48) = 0.23, *p* = .798, η_p_^2^ = .009, no effect of electrode, *F*(4, 96) = 0.48, ε = .39, *p* = .574, η_p_^2^ = .020, and no lag × electrode interaction, *F*(8, 192) = 1.65, ε = .38, *p* = .186, η_p_^2^ = .064, indicating that C1 amplitude did not vary as a function of lag or across electrodes.

Because there was wide individual variability in the magnitude of the T2 identification deficit observed at lag 2 relative to lags 1 and 7 (2–32%), we also examined whether a lag-related C1 effect might vary with AB magnitude. To test this possibility, participants were divided into two groups, based upon a median split of the difference in T2 accuracy between lag 2 and the average of lags 1 and 7: those showing a large AB effect and those showing a small AB effect. A 3 (lag)×5 (electrode) within-subjects ANOVA applied to the large AB effect group revealed no significant main effect of lag, *F*(2, 24) = 1.77, ε = .69, *p* = .204, η_p_^2^ = .128, no effect of electrode, *F*(4, 48) = 0.20, ε = .36, *p* = .744, η_p_^2^ = .017, and no lag × electrode interaction, *F*(8, 96) = 1.26, ε = .30, *p* = .302, η_p_^2^ = .095. The same analysis applied to the small AB effect group also revealed no significant main effect of lag, *F*(2, 26) = 1.97, ε = .68, *p* = .177, η_p_^2^ = .131, no effect of electrode, *F*(4, 52) = 0.89, ε = .41, *p* = .408, η_p_^2^ = .064, and no lag × electrode interaction, *F*(8, 104) = 1.31, ε = .43, *p* = .282, η_p_^2^ = .092. These findings further strengthen the suggestion that C1 amplitude was not modulated by lag.

To ensure that the C1 component reflected neural activity evoked in V1, grand average difference waveforms (collapsed across lags) were subjected to a source localisation using standardized, low-resolution electromagnetic tomography (sLORETA; free academic software publicly available at http://www.uzh.ch/keyinst/loreta.htm). sLORETA uses a minimum norm inverse solution to estimate the cerebral sources of EEG data [48], [49]. The solution space for this method is generated by partitioning the cortical grey matter of the MNI152 template [50] into 6,239 voxels at 5 mm spatial resolution. sLORETA estimates the current density distribution across these voxels most consistent with the electrical activity observed at the scalp electrodes at a specific point in time. Standard electrode positions on the MNI152 scalp were taken from [51]. Figure 3B depicts the sLORETA solution for the grand average difference waveforms at the peak of the C1 component (87 ms post stimulus). Consistent with previous research indicating the C1 component is generated in V1 [11], [12], [16], [43], [52], [53], sLORETA determined that the most active region at this point in time fell within Brodmann area 17 (V1). As can be seen in Figure 3B, sLORETA estimated the activity underlying the C1 component was generated predominantly in the left hemisphere, as would be expected for a visual probe presented in the right visual field. These findings support the suggestion that the C1 component as measured here reflected early activity evoked in V1.

As with the C1, the P1 and N1 components associated with the probe stimulus were identified on the basis of visual inspection of grand average waveform topography maps, and by comparison with the ERP results from Di Russo et al. [12]. The P1 component was measured as the mean amplitude between 100 and 130 ms post stimulus onset at electrodes P7, P5, PO7, PO3, and O1. A 3 (lag)×5 (electrode) within-subjects ANOVA on mean P1 amplitudes revealed no significant main effect of lag, *F*(2, 48) = 0.21, *p* = .814, η_p_^2^ = .009, no effect of electrode, *F*(4, 96) = 1.88, ε = .62, *p* = .153, η_p_^2^ = .073, and no lag × electrode interaction, *F*(8, 192) = 1.15, ε = .47, *p* = .336, η_p_^2^ = .046, indicating that P1 amplitude did not vary as a function of lag or across electrodes. The N1 component was measured as the mean amplitude between 140 and 170 ms post stimulus onset at electrodes F3, F1, Fz, FC3, FC1, and FCz. A 3 (lag)×6 (electrode) within-subjects ANOVA on mean N1 amplitudes revealed a significant main effect of electrode, *F*(5, 120) = 5.18, ε = .53, *p* = .004, η_p_^2^ = .177, indicating that N1 amplitude varied across the six analysed electrodes. However, there was no significant main effect of lag, *F*(2, 48) = 0.15, ε = .74, *p* = .794, η_p_^2^ = .006, and no lag × electrode interaction, *F*(10, 240) = 0.57, ε = .37, *p* = .669, η_p_^2^ = .023, indicating that N1 amplitude did not vary as a function of lag at any of the analysed electrodes. These findings are consistent with previous ERP studies of the AB [17], [18].

## Discussion

We took advantage of the high temporal resolution of the ERP technique to examine early perceptual processing of stimuli presented during the AB. Theoretical models of the AB generally assume it is caused by limitations at post-perceptual stages of processing, and that initial cortical registration of stimuli presented during the AB remains unaffected [54]–[57]. A potential challenge to this assumption has been provided by recent fMRI studies [6], [34], which reported AB-related reductions in neural responses to stimuli presented during the AB in early cortical areas, including V1. However, as visual perception likely reflects both an initial feedforward sweep of information from lower to higher areas of cortex as well as re-entrant feedback from higher back to lower areas [35], [38], [39], the reduced V1 BOLD response observed by Williams et al. [6] and Hein et al. [34] may have resulted from feedback from post-perceptual cortical areas back to V1.

To test this possibility, we examined the integrity of the C1, an ERP component that reflects the initial feedforward sweep of activity through V1 [12], [43], [44], [52], evoked by irrelevant probe stimuli presented during the AB. Although later ERP components have previously been found to be unaffected by the AB [17], [18], the integrity of the C1 has not been investigated until now. Consistent with the typical pattern of behavioural results observed in AB studies [5], [47], participants' T2 accuracy was compromised when it was presented 200 ms after T1, compared to when if followed T1 immediately or was presented 700 ms following T1. In contrast, the amplitude of the C1 component elicited by the irrelevant probe did not vary as a function of lag. Consistent with previous ERP studies of the AB [17], [18], the P1 and N1 components evoked by the probe stimulus were also found to be constant in amplitude across the three lags. Taken together, these findings provide strong support for accounts that posit a post-perceptual locus of the AB, and suggest that the V1 modulation in BOLD responses to T2 reported previously [6], [34] were likely to have arisen from inhibitory feedback from extrastriate areas [54]–[57].

It must be acknowledged that the absence of an effect of lag on early ERP components observed here and in previous studies [17], [18] cannot definitively rule out the possibility of early suppression during the AB. This is because scalp-recorded EEG is only able to measure a small portion of the activity occurring in the brain at any point in time [58]. For neural activity to manifest as voltage changes detectable by electrodes attached to the scalp, millions of neurons must be simultaneously active, and the electromagnetic fields resulting from their activity must be aligned in roughly the same direction. As such, it is possible that modulations of perceptual processing during the AB went undetected in the present study because they occurred in regions of occipital cortex that are inaccessible to ERP measurements. That said, the fact that previous ERP studies have demonstrated effects of spatial attention manipulations on all three of the ERP components examined here [11], [13]–[16], provides a reasonable indication that our measures are sensitive to attention manipulations under some conditions.

Another possibility is that including both correct and incorrect T2 trials in our analyses obscured AB-related effects on early perceptual ERP component. Comparing trials on the basis of whether T2 was correctly identified or missed could potentially reveal modulations of perceptual ERP components that occur only when correct identification of T2 is compromised. Insufficient trial numbers in the present study precluded such an analysis, due to the large number of trials required to obtain reliable ERP measures of the C1 [11], coupled with the relatively high T2 accuracy (∼70% correct for the group at lag 2). However, we believe this option is unlikely to explain our results because previous studies revealed no modulation of the P1 and N1 components on the basis of T2 identification accuracy [18]. Moreover, modulations of the later (post-perceptual) P3 component have been observed even when correct T2 trials were included in the analysis [17], [27], [28], suggesting that ERP measures may be sensitive to AB effects even when conscious awareness of T2 is not compromised.

A third possible alternative explanation for our results stems from the fact that we measured ERP responses to an irrelevant stimulus presented simultaneously with T2, but at a different spatial location, as opposed to directly measuring ERP responses to T2 itself. Processing of an irrelevant stimulus might remain unaffected even when initial perceptual processing of T2 (or stimuli presented at the same location as T1) is compromised during the AB. Several lines of evidence argue against this possibility. First, many of studies have reported robust (and even enhanced) behavioural AB deficits when T1 and T2 are presented at different spatial locations (see [59]). Second, Williams et al. [6] found that BOLD responses were suppressed during the AB not only in the region of V1 corresponding to the spatial location of T2, but also in regions of V1 corresponding to simultaneously presented distractor stimuli at locations elsewhere in the visual field. These findings suggest that any AB-related effects should be present across the entire visual field, and not be restricted to either T2 or the location of T1. That said, these results are not entirely conclusive on this issue because stimuli in these studies were never presented in completely task-irrelevant spatial locations as they were in the present work. For this reason, it would be useful for future studies to establish how C1 amplitudes during the AB vary as a function of stimulus relevance and the relevance of the probe location.

A final point concerns our choice of target task and stimuli. Although identification of alphanumeric characters is a common choice for target tasks in behavioural AB studies, previous fMRI studies that have found AB-related modulation of activity in early visual cortex [6], [34] have used relatively simple stimuli that could be discriminated solely on the basis of orientation (e.g., the orientation of a grating). As such, one potential explanation for the lack of modulation of the C1 component observed here is that changes in early visual cortex activity emerge only when the T2 task involves discriminating basic features processed in early visual cortex, such as orientation. This suggestion follows from theoretical models which argue that the locus of attentional effects can vary flexibly depending on the processing stage most heavily taxed by the current task (e.g., [60]). This possibility could be addressed in a future study by having participants judge the orientation of a grating, rather than an alphanumeric character, as the T2 task. It is worth noting, however, that both simple and complex stimuli yield similar modulations of later parietal activity [29], [34]. Moreover, it is important to note that the T2 task used by Williams et al. [6] consisted of localizing an ‘X’ presented amongst three ‘+’ signs. While this task could be accomplished by judging orientation alone, it seems likely that semantic representations available for both targets and distractors were also accessed by observers during the task.

In summary, consistent with previous ERP studies of the AB [17], [18], the present findings suggest that stimuli presented during the AB undergo a similar amount of early perceptual processing as stimuli presented outside the AB, at least in situations with alphanumeric targets. Our study extended these previous findings by examining the C1 component, which is the only component examined thus far in the literature that is known to reflect the initial feedforward sweep of information through V1 [12]. The current finding of uniform C1 amplitudes across lags suggests that fMRI evidence for reduced V1 activity during the AB [6], [34] is likely to reflect modulations of re-entrant feedback signals [35]–[39] from higher cortical areas back to V1, after initial registration of T2. Such involvement of re-entrant feedback in top-down modulation of activity in early visual cortex is not a novel proposal, and has been advanced previously to explain the influence of spatial attention on activity in early visual areas [41], [42], [53], [61], [62]. This proposal is well supported by complimentary ERP and fMRI evidence that spatial attention-related modulations of activity in V1 are preceded by modulations in higher areas of visual cortex [41], [42], [53], [61], [62]. The ERP results reported here indicate similar feedback mechanisms may exist for bottlenecks in the temporal allocation of attention, and support theoretical accounts of the AB that postulate a role for re-entrant processing in modulating the effect [36].
